# Participation limitations as a transdiagnostic feature in serious mental illness: confirmatory modeling

**DOI:** 10.1192/j.eurpsy.2024.325

**Published:** 2024-08-27

**Authors:** J. Bar Yosef, L. Lipskaya-Velikovsky

**Affiliations:** ^1^Occupational Therapy, Clalit, Afula; ^2^The school of occupational therapy, Hebrew University of Jerusalem, Jerusalem, Israel

## Abstract

**Introduction:**

Participation in daily life occupations of personal and community meaning is an important component of health and recovery from mental illness. Limitations in participation were found to be a hallmark of serious mental illness (SMI). Still, previous research has mainly focused on objective dimensions of participation, largely neglecting the subjective aspects that hold particular relevance for health outcomes. Next, participation was addressed by specific diagnoses, approach which is divergent from the recovery model, a transdiagnostic approach and clinical practice. Hence, further research into participation is warranted to broaden our understanding.

**Objectives:**

We investigated objective and subjective patterns of participation across a range of SMI diagnoses to delineate differences, and to identify personal and illness-related factors associated with participation dimensions.

**Methods:**

A secondary analysis of cross-sectional studies (N=14). The analysis included data from 489 men (40.7%) and women (59.3%) diagnosed with one of 4 SMI conditions: psychotic, affective (AD), post-traumatic (PTSD) or personality (PD) disorders. The participants were aged 18 to 60 (M=34.41; SD=10.9) and were in contact with intensive mental health services. All participants completed the Adult Subjective Assessment of Participation (ASAP), which comprised participation intensity, diversity, satisfaction and enjoyment, and standard evaluations of cognitive functioning, symptom severity, and functional capacity. Z-scores were calculated for independent variables to enable comparison. Demographic and illness-related (IR) information was also collected.

**Results:**

Frequency of participation was found to be significantly different between diagnostic groups, but not participation diversity, enjoyment and satisfaction. Participation diversity was altered by range of demographic variables (5.26<F<10.6, p<.01, 0.3<<0.4) while participation frequency differs by employment status (t (485) =-2.84, p<0.05, Cohen’s d=0.25). No differences were found between groups in symptoms’ severity. Regression analysis indicates that cognition, functional capacity and employment status explain in a significant way integrated index of objective participation (*χ^2^* =47.52, p<0.001). For the subjective dimension, the logistic regression was not found statistically significant (*χ^2^* =20.99, p=0.51).

**Conclusions:**

Limitations in diversity, enjoyment and satisfaction with participation, were demonstrated to be a transdiagnostic feature in SMI. Objective participation dimensions can be explained with demographic, personal and illness related factors, while modeling of subjective dimensions should be further investigated.

**Disclosure of Interest:**

None Declared

